# Regional and racial variations in the utilization of endoscopic retrograde cholangiopancreatography among pancreatic cancer patients in the United States

**DOI:** 10.1002/cam4.2225

**Published:** 2019-05-14

**Authors:** Anna Tavakkoli, Amit G. Singal, Akbar K. Waljee, James M. Scheiman, Caitlin C. Murphy, Sandi L. Pruitt, Lei Xuan, Richard S. Kwon, Ryan J. Law, Grace H. Elta, Phyllis Wright‐Slaughter, Thomas S. Valley, Nisa Kubiliun, Hari Nathan, Joel H. Rubenstein, B. Joseph Elmunzer

**Affiliations:** ^1^ Division of Digestive and Liver Diseases, Department of Internal Medicine University of Texas Southwestern Dallas Texas; ^2^ VA Center for Clinical Management Research VA Ann Arbor Health Care System Ann Arbor Michigan; ^3^ Division of Gastroenterology and Hepatology, Department of Internal Medicine Michigan Medicine Ann Arbor Michigan; ^4^ Michigan Integrated Center for Health Analytics and Medical Prediction (MiCHAMP) Ann Arbor Michigan; ^5^ Division of Gastroenterology University of Virginia Charlottesville Virginia; ^6^ Department of Clinical Sciences University of Texas Southwestern Dallas Texas; ^7^ Institute for Health Policy and Innovation University of Michigan Ann Arbor Michigan; ^8^ Division of Pulmonary and Critical Care Medicine, Department of Internal Medicine Michigan Medicine Ann Arbor Michigan; ^9^ Department of Surgery Michigan Medicine Ann Arbor Michigan; ^10^ Division of Gastroenterology and Hepatology, Department of Medicine Medical University of South Carolina Charleston South Carolina

**Keywords:** disparities, ERCP, obstructive jaundice, pancreatic cancer

## Abstract

**Background:**

Pancreatic cancer is projected to become the second leading cause of cancer‐related deaths by 2030. Endoscopic retrograde cholangiopancreatography (ERCP) is recommended as first‐line therapy for biliary decompression in pancreatic cancer. The aim of our study was to characterize geographic and racial/ethnic disparities in ERCP utilization among patients with pancreatic cancer.

**Methods:**

Retrospective cohort study using the US Surveillance, Epidemiology, and End Results (SEER)‐Medicare database to identify patients diagnosed with pancreatic cancer from 2003‐2013. The primary outcome was receipt of ERCP, with or without stent placement, vs any non‐ERCP biliary intervention.

**Results:**

Of the 36 619 patients with pancreatic cancer, 37.5% (n = 13 719) underwent an ERCP, percutaneous drainage, or surgical biliary bypass. The most common biliary intervention (82.6%) was ERCP. After adjusting for tumor location and stage, Blacks were significantly less likely to receive ERCP than Whites (aOR 0.84, 95% CI 0.72, 0.97) and more likely to receive percutaneous transhepatic biliary drainage (PTBD) (aOR 1.38, 95% CI 1.14, 1.66). Patients in the Southeast and the West were more likely to receive ERCP than those in the Northeast (Southeast aOR 1.21, 95% CI 1.04, 1.40; West aOR 1.16, 95% CI 1.01, 1.32).

**Conclusion:**

Racial/ethnic and geographic disparities in access to biliary interventions including ERCP exist for patients with pancreatic cancer in the United States. Our results highlight the need for further research and policies to improve access to appropriate biliary intervention for all patients.

## 1 INTRODUCTION

Pancreatic cancer is one of the deadliest malignant neoplasms, with a five‐year survival of only 7%.^1^ The incidence of pancreatic cancer has increased over the past decade, and it is projected to become the second leading cause of cancer‐related deaths by 2030 in the United States.^2^, ^3^ The poor prognosis of pancreatic cancer can be attributed in part to the large proportion of patients that present at an advanced stage which precludes surgical resection.

Biliary decompression is often required in advanced pancreatic cancer for symptomatic relief and to allow neoadjuvant chemotherapy in patients with borderline resectable tumors.^4^, ^5^ Endoscopic retrograde cholangiopancreatography (ERCP) plays a critical role in the management of obstructive jaundice among pancreatic cancer patients. When compared to other biliary decompression interventions, such as percutaneous transhepatic biliary drainage (PTBD) or surgical biliary bypass (SBB), ERCP is associated with fewer adverse events, shorter length of stay, decreased hospital costs, and improved quality of life scores.^6^, ^7^ Studies appraising the utilization of biliary decompression interventions can inform strategies to increase ERCP access and appropriate utilization.

Despite the importance of ERCP in the treatment of pancreatic cancer, it remains unknown whether racial/ethnic or regional disparities exist for the use of ERCP. These disparities have been described in access to other pancreatic cancer treatments. Black patients are less likely than Whites to be referred for curative surgery or chemotherapy, even after adjusting for tumor stage.^8^, ^9^, ^10^, ^11^, ^12^ Furthermore, patients with early stage pancreatic cancer in the Northeast are more likely to be referred for surgical resection than those in the Southeast, Midwest, and Pacific West.^9^ Given the findings from prior studies, we hypothesized that there are racial/ethnic and geographic disparities in access to biliary interventions, including ERCP, such as Black patients being less likely to receive ERCP as an initial biliary intervention. The aim of our study was to characterize geographic and racial/ethnic disparities in ERCP utilization among patients with pancreatic cancer.

## 2 METHODS

### 2.1 Data sources

We conducted a retrospective cohort study using the Surveillance, Epidemiology, and End Results (SEER)‐Medicare database. The SEER‐Medicare database contains data on patient demographics, clinical characteristics, tumor location and staging, diagnostic and therapeutic treatments, and overall survival for all included patients.^13^ The SEER program collects data from 17 cancer registries and represents roughly 27% of the population of the United States, while the Medicare database contains health insurance claims for approximately 97% of the population that is 65 years or older.^13^ Institutional Review Board (IRB) exemption was obtained to review previously collected data (HUM00128282).

### 2.2 Study sample

We included patients diagnosed with pancreatic cancer from 2003 to 2013. Pancreatic cancer histology was based on the International Classification of Diseases for Oncology (ICD‐O‐3) codes (Table S1). Biliary interventions evaluated in our study included ERCP, PTBD, and SBB. Patients were excluded if they had a history of other cancer, histology other than adenocarcinoma, or if their pancreatic cancer diagnosis was made at time of death or on autopsy (Figure S1). We excluded patients with multiple biliary interventions on the same date due to unclear order of procedures and concerns for coding errors since this is unlikely to happen in clinical practice. Patients with biliary interventions greater than 2 months before their diagnosis were also excluded given this was more likely related to reasons other than pancreatic cancer. We evaluated patient enrollment in non‐health maintenance organizations (HMOs), Medicare Part A and Medicare Part B. Patients were required to have continuous enrollment in Medicare Part A and B coverage, without concomitant enrollment in an HMO, for at least two months prior to their pancreatic cancer diagnosis and up to 12 months after their diagnosis or to death. This was because some of their claims may be captured by HMOs rather than by Medicare. (Figure S1). The MEDPAR and outpatient files were used to identify diagnosis and procedural codes using International Classification of Diseases (ICD)‐9 codes, the American Medical Association Common Procedure Terminology (CPT) codes, and the health care common procedures codes (HCPCS) (Table S1).^9^, ^11^, ^14^, ^15^, ^16^

### 2.3 Study variables

All variables used in the study were available in SEER‐Medicare. Sex and race/ethnicity were obtained from the SEER file. Race/ethnicity, based on SEER designation, was classified as White, Black, Asian, Hispanic, Native American, other, or unknown. Patient age was based on the Medicare birth month, day, and year. The age was calculated as the age at the date of diagnosis. Date of diagnosis was based on SEER designated date of pancreatic cancer diagnosis. The SEER designated date of diagnosis has been shown to have a nearly 90% agreement with the first Medicare claim with a cancer diagnosis.^17^ Charlson comorbidity index (CCI) was calculated using the MEDPAR and outpatient claims one year prior to their pancreatic cancer diagnosis.

Tumor stage was defined using American Joint Committee of Cancer (AJCC) staging, sixth edition. However, AJCC staging was available in SEER‐Medicare from 2004 to present. Tumor stage for patients diagnosed in 2003 was considered missing for purposes of our analysis. Location of pancreatic tumor, based on the SEER primary site, was designated as head of pancreas, body/tail of pancreas, or unknown.

SEER regions were divided into Northeast (Connecticut and New Jersey), Southeast (Atlanta, greater Georgia, rural Georgia, Kentucky, and Louisiana), Midwest (Detroit and Iowa), and the West (San Francisco, Los Angeles, San Jose, greater California, New Mexico, Seattle, Utah, and Hawaii).^18^

Clinical characteristics were also obtained from SEER‐Medicare. From the MEDPAR files, we were able to determine whether a patient required ICU level care in either the general, medical, or surgical ICU and the date of the admission to the ICU. Patients were determined to require ICU level care if the admission date was after the time of their diagnosis (if no biliary intervention was performed) or at/after the time of their biliary intervention. ICD‐9 codes from the MEDPAR files were used to identify patients with jaundice, cholangitis, or gastric outlet obstruction during any admission after the time of their diagnosis but prior to a biliary intervention, if they received one. SEER has recorded whether patients have received site‐specific surgery with classifications to the type of surgery they received, including a Whipple. Patients were considered to have received a Whipple procedure based on this SEER designation of receiving site specific surgery. However, the date of this procedure was not recorded in the SEER file. Last, ICD‐9 codes and CPT codes were used to identify if a patient received an ERCP, PTBD, or SBB and the date of their procedure.

### 2.4 Outcomes

Our primary outcome was receipt of ERCP, with or without stent placement, vs a non‐ERCP biliary intervention (ie PTBD or SBB). Patients without any biliary decompression were excluded. We also excluded patients with Whipple resection because we could not accurately determine if the biliary intervention preceded or post‐dated the surgery.

We included any biliary intervention that occurred within two months prior to pancreatic cancer diagnosis or anytime after diagnosis because: (a) biliary decompression can be achieved prior or concurrent to confirming a diagnosis of pancreatic cancer and (b) pancreatic cancer codes may be delayed after a diagnosis has been confirmed.^19^ A two‐month window was determined a priori based on expert opinion; the appropriateness of this cut‐off was confirmed as there was a stepup in the frequency of biliary interventions two months prior to diagnosis of pancreatic cancer in our dataset.

### 2.5 Statistical analysis

We first characterized the proportions of patients who received ERCP, non‐ERCP biliary drainage, and no intervention. For our analysis, we identified correlates of ERCP receipt using Student *t* test and chi‐square test for continuous and categorical variables, respectively. The Cochrane‐Armitage test for trend was used to evaluate receipt of ERCP by year. Stepwise forward regression was used to identify covariates of interest which were ultimately used in our multivariable logistic model. The covariates in our analysis were: race, gender, age at the time of diagnosis, SEER region, location of tumor (head, body, tail of pancreas), AJCC tumor stage, sixth edition (Stage I‐Stage IV), year of diagnosis (continuous variable), requirement of ICU stay, CCI, and presence of cholangitis, gastric outlet obstruction, or jaundice. Analyses were conducted using Stata 15.0 (Stata Corp, College Station, TX).

## 3 RESULTS

### 3.1 Study sample and patient demographics

Of 83 164 potentially eligible patients, we excluded patients with a history of other cancers (n = 17 348), histology other than adenocarcinoma (n = 4065), initial diagnosis of pancreatic cancer on death certificate or autopsy (n = 2392), and patients with non‐continuous Part A/B coverage or coverage by an HMO during the study period (n = 19 522). Of the remaining 39 837 patients, we excluded 837 patients who had no Medicare Part A or B claims, 1752 patients who had a biliary intervention greater than 2 months prior to their diagnosis, and 629 patients who had more than one procedure on the same date. Overall, there were 36 619 eligible patients with pancreatic cancer (Figure S1).

Demographics of included patients are shown in Table 1. Most patients were White and between the age of 65 and 80 years old. Nearly half of the patients presented with tumors in the head of the pancreas, and over 40% had stage IV disease at the time of diagnosis. Only 11% of patients had jaundice and less than 5% of patients received ICU care after pancreatic cancer diagnosis. Among the more than 1400 patients who required ICU level care after their diagnosis, the majority (58%) received a biliary intervention (Table 1).

**Table 1:** Baseline characteristics of patient cohort

	Total number (%)	Biliary intervention
Total number	36 619	13 719 (37.5%)
Biliary intervention		
ERCP	—	11 333 (82.6%)
Percutaneous drainage	—	1210 (8.8%)
Surgical bypass	—	1176 (8.6%)
Gender		
Male	16 376 (44.7%)	6037 (44.0%)
Female	20 243 (55.3%)	7682 (56.0%)
Race		
White	29 365 (80.2%)	10 973 (80.0%)
Black	4089 (11.2%)	1561 (11.4%)
Asian	1308 (3.6%)	476 (3.5%)
Hispanic	760 (2.1%)	311 (2.3%)
Native American	139 (0.4%)	47 (0.3%)
Other	958 (2.6%)	351 (2.6%)
Year of diagnosis		
2003	3319 (9.1%)	1381 (10.1%)
2004	3368 (9.2%)	1399 (10.2%)
2005	3387 (9.3%)	1331 (9.7%)
2006	3491 (9.5%)	1296 (9.5%)
2007	3431 (9.4%)	1266 (9.2%)
2008	3542 (9.7%)	1293 (9.4%)
2009	3425 (9.4%)	1267 (9.2%)
2010	3462 (9.5%)	1225 (8.9%)
2011	3390 (9.3%)	1214 (8.9%)
2012	3279 (9.0%)	1158 (8.4%)
2013	2525 (6.9%)	889 (6.5%)
Age at diagnosis		
<65	3254 (8.9%)	1027 (7.5%)
65‐69	6565 (17.9%)	2514 (18.3%)
70‐79	13 800 (37.7%)	5305 (38.7%)
80‐89	10 690 (29.2%)	4112 (30.0%)
>90	2310 (6.3%)	761 (5.6%)
Location of pancreatic tumor		
Head of pancreas	17 658 (48.2%)	9914 (72.3%)
Body/tail	7949 (21.7%)	856 (6.2%)
Unknown	11 012 (20.1%)	2949 (21.5%)
SEER demographic		
Northeast	7825 (21.4%)	2975 (21.7%)
Southeast	8786 (24.0%)	3325 (24.2%)
Midwest	4440 (12.1%)	1596 (11.6%)
West	15 568 (42.5%)	5823 (42.4%)
Stage of disease (AJCC), sixth edition^a^		
Stage I	2308 (6.3%)	1092 (8.0%)
Stage II	7257 (19.8%)	3369 (24.6%)
Stage III	2459 (6.7%)	1252 (9.1%)
Stage IV	15 509 (42.4%)	4177 (30.5%)
Unknown	5767 (15.8%)	2448 (17.8%)
Charlson comorbidity index		
0	10 835 (29.6%)	4103 (29.9%)
1	7309 (20.0%)	2796 (20.4%)
2	9647 (26.3%)	3810 (27.8%)
Jaundice		
Yes	4041 (11.0%)	2937 (21.4%)
No	32 578 (89.0%)	10 782 (78.6%)
Cholangitis		
Yes	369 (1.0%)	296 (2.2%)
No	36 250 (99.0%)	13 423 (97.8%)
ICU stay after diagnosis		
Yes	1420 (3.9%)	823 (6.0%)
No	35 199 (96.1%)	12 896 (94.0%)
Gastric outlet obstruction		
Yes	267 (0.7%)	181 (1.3%)
No	36 325 (99.3%)	13 538 (98.7%)

a Does not include 2003 diagnoses.

In total, 13 719 patients underwent a biliary decompressive intervention. The most common biliary decompressive intervention in this cohort was ERCP (82.6%) (Table 1). The remainder of patients underwent PTBD (8.8%) or SBB (8.6%) (Table 1). There was a decrease in the overall use of biliary interventions from 2003 to 2013 (Table 1). The majority of patients who underwent a biliary intervention had a mass in the head of the pancreas (72.3%) (Table 1). Among the 13 000 patients who underwent a biliary intervention, 30.5% had stage IV disease (Table 1).

### 3.2 Receipt of ERCP as initial biliary intervention

Compared to patients who had a non‐ERCP biliary intervention, a greater proportion of patients who underwent an ERCP were White, have a mass in the pancreatic head, have early stage cancer, and present with jaundice or cholangitis (Table 2). While the use of ERCP decreased from 2003 to 2013, the use of non‐ERCP interventions also decreased (Table 2). A fewer proportion of patients who received an ERCP required a stay in the ICU as compared to those patients who underwent a non‐ERCP intervention (Table 2). There was no statistically significant difference in the CCI between patients who underwent ERCP vs a non‐ERCP intervention (Table 2).

**Table 2:** Characteristics of patients with ERCP vs non‐ERCP biliary intervention

	ERCP (n, %)	Non‐ERCP intervention (n, %)	*P*‐value
Total number (n = 13 719)	11 333 (82.6%)	2386 (17.4%)	
Gender			
Male	4949 (43.7%)	1088 (45.6%)	0.084
Female	6384 (56.3%)	1298 (54.4%)	
Race			
White	9135 (80.6%)	1838 (77.0%)	0.01
Black	1244 (11.0%)	317 (13.3%)	
Asian	379 (3.3%)	97 (4.1%)	
Hispanic	251 (2.2%)	60 (2.5%)	
Native American	36 (0.3%)	11 (0.5%)	
Other	288 (2.5%)	63 (2.6%)	
Year of diagnosis			
2003	1081 (9.5%)	300 (12.6%)	0.68^b^
2004	1147 (10.1%)	252 (10.6%)	
2005	1060 (9.4%)	271 (11.4%)	
2006	1037 (9.2%)	259 (10.9%)	
2007	1048 (9.3%)	218 (9.1%)	
2008	1083 (9.6%)	210 (8.8%)	
2009	1034 (9.1%)	233 (9.8%)	
2010	1034 (9.1%)	191 (8.0%)	
2011	1035 (9.1%)	179 (7.5%)	
2012	1006 (8.9%)	152 (6.4%)	
2013	768 (6.8%)	121 (5.1%)	
Age at diagnosis			
<65	834 (7.4%)	193 (8.1%)	<0.001
65‐69	2023 (17.9%)	491 (20.6%)	
70‐79	4348 (38.4%)	957 (40.1%)	
80‐89	3474 (30.7%)	638 (26.7%)	
>90	654 (5.8%)	107 (4.5%)	
Location of pancreatic tumor			
Head of pancreas	8245 (72.8%)	1669 (70.0%)	0.008
Body/tail	681 (6.0%)	175 (7.3%)	
Unknown	2407 (21.2%)	542 (22.7%)	
SEER demographic			
Northeast	2425 (21.4%)	550 (23.1%)	0.063
Southeast	2788 (24.6%)	537 (22.5%)	
Midwest	1302 (11.5%)	294 (12.3%)	
West	4818 (24.5%)	1005 (42.1%)	
Stage of disease (AJCC), sixth edition^a^			
Stage I	959 (9.4%)	133 (6.4%)	<0.001
Stage II	2831 (27.6%)	538 (25.8%)	
Stage III	986 (9.6%)	266 (12.8%)	
Stage IV	3368 (32.9%)	809 (38.8%)	
Unknown	2108 (20.6%)	340 (16.3%)	
Charlson comorbidity index			
0	3402 (38.2%)	701 (38.9%)	0.71
1	2339 (26.3%)	457 (25.4%)	
≥2	3166 (35.6%)	644 (35.7%)	
Jaundice			
Yes	2612 (23.1%)	325 (13.6%)	<0.001
No	8721 (77.0%)	2061 (86.4%)	
Cholangitis			
Yes	271 (2.4%)	25 (1.1%)	<0.001
No	11 062 (97.6%)	2361 (99.0%)	
ICU Stay after diagnosis			
Yes	413 (3.6%)	410 (17.2%)	<0.001
No	10 920 (96.4%)	1976 (82.8%)	
Gastric outlet obstruction			
Yes	146 (1.3%)	35 (1.5%)	0.49
No	11 187 (98.7%)	2351 (98.5%)	

a Does not include 2003 diagnoses.

b *P*‐value represents test for trend.

There were racial/ethnic and geographic disparities in receipt of ERCP as the initial biliary intervention after adjusting for tumor stage and clinical presentation (Table 3). Blacks were significantly less likely to receive an ERCP compared to Whites (aOR 0.84, 95% CI 0.72, 0.97) (Table 3). However, Blacks were more likely to receive PTBD compared to Whites (aOR 1.38, 95% CI 1.14‐1.66) (results not shown). Patients in the Southeast and the West were more likely to receive ERCP compared to those in the Northeast (Table 3).

**Table 3:** Correlates for receipt of ERCP as initial biliary intervention vs non‐ERCP interventions

	Unadjusted (OR, 95% CI)	Adjusted (aOR, 95% CI)
ERCP		
Race		
White	Ref	Ref
Black	**0.79 (0.69, 0.90)**	**0.84 (0.72, 0.97)**
Asian	**0.79 (0.63, 0.99)**	0.78 (0.61, 1.01)
Hispanic	0.84 (0.63, 1.12)	0.82 (0.60, 1.12)
Native American	0.66 (0.33, 1.30)	0.68 (0.33, 1.44)
Gender		
Male	Ref	Ref
Female	1.08 (0.99, 1.18)	1.05 (0.95, 1.16)
Age at diagnosis		
<65	Ref	Ref
65‐69	0.95 (0.79, 1.15)	0.92 (0.75, 1.12)
70‐79	1.05 (0.89, 1.25)	0.98 (0.82, 1.19)
80‐89	**1.26 (1.05, 1.51)**	1.08 (0.88, 1.32)
>90	**1.41 (1.09, 1.83)**	1.21 (0.90, 1.61)
SEER region		
Northeast	Ref	Ref
Southeast	**1.18 (1.03, 1.34)**	**1.21 (1.04, 1.40)**
Midwest	1.00 (0.86, 1.17)	0.99 (0.84, 1.18)
West	1.09 (0.97, 1.22)	**1.16 (1.01, 1.32)**
Location of tumor		
Head of pancreas	Ref	Ref
Body/tail	**0.79 (0.66, 0.94)**	0.89 (0.73, 1.08)
Stage of disease (AJCC)^a^		
Stage I	Ref	Ref
Stage II	**0.73 (0.60, 0.89)**	**0.75 (0.61, 0.92)**
Stage III	**0.51 (0.41, 0.64)**	**0.55 (0.44, 0.70)**
Stage IV	**0.58 (0.47, 0.70)**	**0.63 (0.51, 0.78)**
Charlson comorbidity index		
0	Ref	—
1	1.05 (0.93, 1.20)	—
≥2	1.01 (0.90, 1.14)	—
Year of diagnosis		
Per 1 year	**1.06 (1.03, 1.07)**	**1.05 (1.03, 1.07)**
Jaundice		
No	Ref	Ref
Yes	**1.90 (1.68, 2.15)**	**1.68 (1.47, 1.92)**
Cholangitis		—
No	Ref	—
Yes	**2.31 (1.53, 3.49)**	—
ICU stay after diagnosis		
No	Ref	Ref
Yes	**0.18 (0.16, 0.21)**	**0.19 (0.16, 0.22)**
Gastric outlet obstruction		
No	Ref	—
Yes	0.88 (0.60, 1.27)	—

Bolded values are statistically significant.

a Does not include 2003 diagnoses.

## 4 DISCUSSION

In this analysis of population‐based data, we found racial/ethnic and geographic disparities in receipt of biliary interventions, including ERCP, among patients with pancreatic cancer. Black pancreatic cancer patients were less likely to receive an ERCP, which is the preferred route of biliary decompression, and more likely to receive PTBD. Patients in the Northeast were less likely to undergo ERCP as compared to those in the Southeast and West. These findings highlight the presence of inequitable utilization of endoscopic procedures that have been shown to play a critical role in the management of pancreatic cancer.

ERCP is recommended as the initial biliary decompressive intervention for patients who present with biliary obstruction due to a pancreatic head mass.^20^ Our results are encouraging in that they demonstrate that the large majority (~83%) of patients who underwent a biliary intervention due to pancreatic cancer received the optimal treatment modality—ERCP. However, our findings also demonstrate that approximately one in six pancreatic cancer patients received PTBD or SBB, with Black patients being less likely than Whites to receive an ERCP. The primary driver of non‐ERCP interventions is unclear at this time. While a growing body of literature, including randomized controlled trials and “real world” cohort studies, have shown that endoscopic biliary drainage is associated with lower adverse events, shorter length of hospitalization, lower costs, and better quality of life scores, our study demonstrates that disparities in access to ERCP across racial/ethnic and geographic cohorts remain.^6^, ^7^ Further studies are needed to determine if these differences are related to accessibility of treatment, local expertise, or regional practice variations in an effort to bridge the gap in care among pancreatic cancer patients.

Minority populations have been found to be especially vulnerable to the inequitable distribution of healthcare across America.^9^, ^21^, ^22^ While factors such as tumor biology may account for some of the differences in cancer related mortality among minority groups, existing literature highlights racial and ethnic disparities in the receipt of cancer treatments, such as Whipple surgery or referrals for chemotherapy.^10^, ^11^, ^12^, ^21^ The findings from our study add to the growing body of literature highlighting disparate receipt of oncological care among Blacks and also highlight that these inequities may exist in the delivery of endoscopic procedures important in the care of pancreatic cancer patients. While differences in access to ERCP may play a central role in these disparities, examining other factors, such as patient preferences, physician preferences, or local expertise are important next steps to understanding the findings from our study.

We also noted regional variations in receipt of ERCP across the United States. Prior studies evaluating receipt of surgery for early‐stage pancreatic cancer found patients in the Northeast were more likely to be referred for curative surgery, possibly due in part to the concentration of high volume, tertiary care centers in the Northeast that specialize in hepato‐pancreato‐biliary surgery.^9^ In our study, we found patients in the Northeast were less likely to receive an ERCP as compared to non‐ERCP biliary interventions if decompression was required. Regional variations in expertise and care could be potential explanations. For example, since patients in the Northeast are more likely to undergo surgery for early‐stage pancreatic cancer, those who are deemed unresectable in the operating room may be receiving SBB prior to closure, reducing the fraction of ERCP treated patients. However, we cannot exclude the possibility of confounding by unmeasured factors. Although existing data describe a >90% success rate in selective biliary cannulation in pancreatic cancer, and the findings from our study show that over 85% of patients received an initial ERCP for biliary decompression, our results suggest that treatment varies throughout the United States.^20^ Future researches should focus on how those disparities may influence clinical outcomes and how access or local expertise in care may change the procedure a patient receives.

Interestingly, almost 45% of our patient population underwent a biliary decompressive intervention although only 11% of patients had an ICD‐9 code for jaundice. This could be due to miscoding or lack of coding for jaundice. Given that the vast majority of ERCPs are performed in a hospital based setting, as opposed to an ambulatory surgical center, the decision was made to use the MEDPAR and outpatient claims files only, rather than including claims from carrier claims files as well. This also prevented double counting of outpatient procedures that may be listed in both the outpatient claims file and carrier claims files. However, carrier claims files include claims data by non‐institutional providers, such as physician assistants, and this could account for a lower than expected percentage of patients with jaundice.

Our study has some important limitations, including those inherent to using insurance claims. First, while linkage of SEER data with Medicare claims data increases available clinical information, we cannot exclude the possibility of residual confounding in multivariable analyses showing racial/ethnic and geographic disparities.^13^, ^23^ Importantly, we were unable to determine necessity for biliary decompression, especially given that over 50% of patients did not undergo a biliary intervention, which may have been appropriate given lack of symptoms, laboratory abnormalities, or significant comorbidities. Second, SEER‐Medicare does not include the entire US and is limited to only the older population. Third, we cannot exclude the possibility of miscoding for important variables such as race/ethnicity, diagnosis, or procedural codes. Last, there was a downtrend in the number of ERCPs performed over our study and especially in 2013. However, this trend has been observed in prior studies evaluating the trend of ERCP in the US.^24^, ^25^

To the best of our knowledge, this is the first population‐based study to examine variations in access to ERCP across the United States. The findings suggest that utilization of ERCP varies according to race/ethnicity and geographic location in the United States. Understanding the clinical implications and factors that play a role in these gaps will be important to improving quality cancer care for all patients with pancreatic cancer.

## CONFLICT OF INTEREST

The authors disclose no conflicts.

## AUTHOR CONTRIBUTIONS

AT contributed draft manuscript, developed study concept and design, acquired, analyzed, and interpreted data, obtained funding. AGS and AKW developed study concept and design, acquired, analyzed, and interpreted data. JMS developed study concept and design. CM, SLP, and LX involved in statistical analysis. RSK, RJL, and GHE contributed to analysis and interpretation of data. PWS involved in administrative support. TSV contributed to statistical analysis, analysis and interpretation of data. NK involved in critical revision of the manuscript for important intellectual content. HN contributed to technical support, statistical analysis, critical revision of the manuscript for important intellectual content. JHR developed study concept and design. BJE developed study concept and design, acquired, analyzed, and interpreted data. All authors critically reviewed the manuscript for important intellectual concepts.
