# Supplementary material for: Regional and racial variations in the utilization of endoscopic retrograde cholangiopancreatography among pancreatic cancer patients in the United States
Source: Cancer Med. 2019 May 14;8(7):3420–7. doi: 10.1002/cam4.2225 (PMC6601581; doi:10.1002/cam4.2225)
Supplement: Supplementary file 1 [file CAM4-8-3420-s001.pdf]

Supplemental Content

Supplemental Figure 1: Flowchart of patient cohort

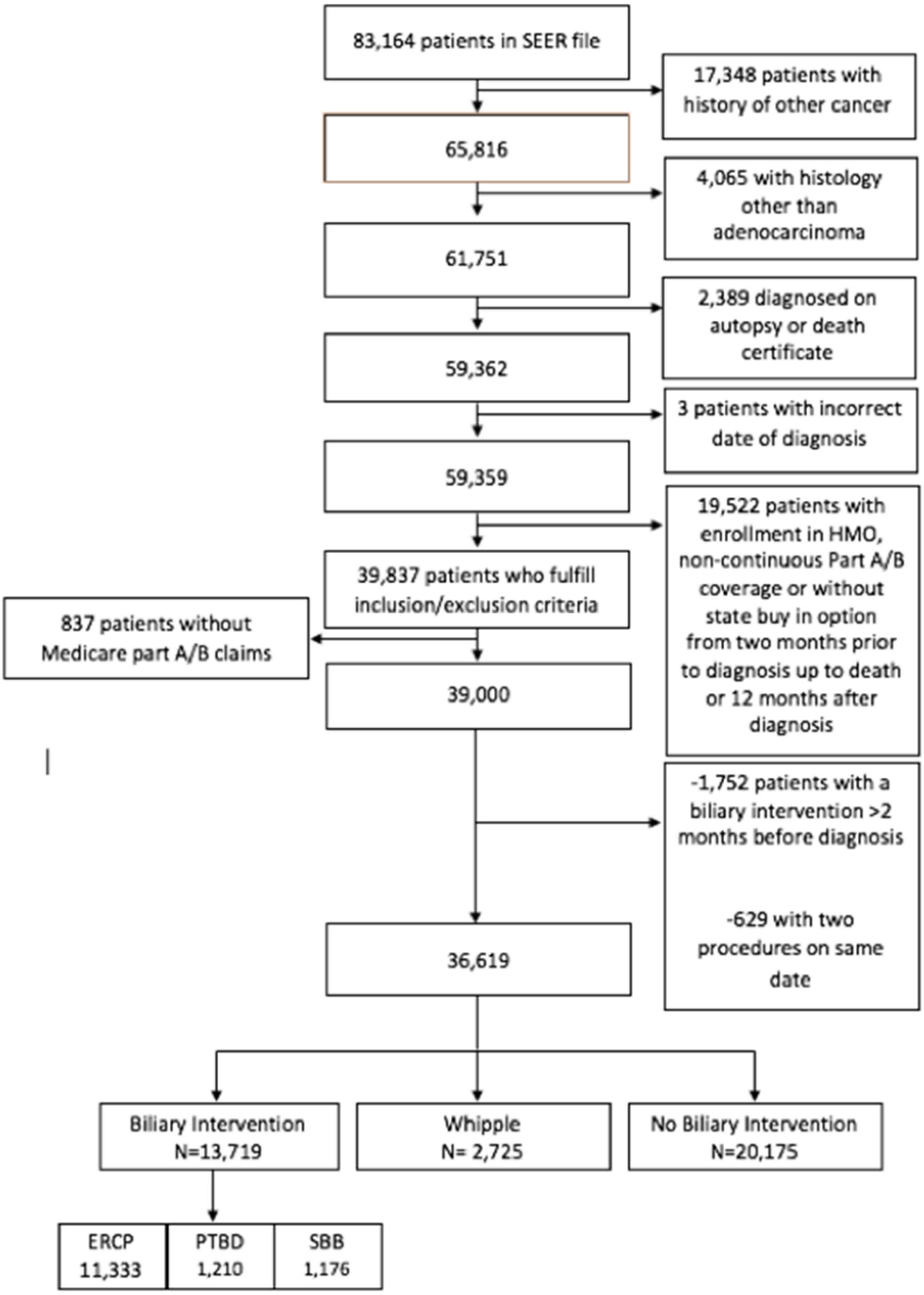

Supplemental Table 1: Medicare and SEER Billing Codes

**Variables**	**Codes**
Pancreatic Adenocarcinoma (ICD-0-3 codes for histology of pancreatic cancer)	8000-5, 8010-15, 8020-22, 8050-55, 8140, 8211, 8230, 8260-63, 8440, 8450-53, 8470-71, 8480-81, 8500, 8503, 8521, 8570
ERCP (ICD-9 and CPT codes for procedures)	43260-65, 4371-72, 74328, 74329, 74330, 5110, 5111, 5184- 87, 5199, 5213, 5293, 43267-69, 43274, 43276, 9705, C1874-76
Jaundice (ICD-9)	7824
Cholangitis (ICD-9)	5761
Gastric Outlet Obstruction (ICD-9)	5370

Supplemental Table 2: Correlates for receipt of ERCP by race (black vs. non-black)

	Black (n, %)	Non-Black (n, %)	p-value
Total Number	1,561 (11.4%)	12,158 (88.6%)	---
Biliary Intervention			
ERCP	1,244 (79.7%)	10,089 (83.0%)	0.001
Non-ERCP	317 (20.3%)	2,069 (17.0)	
Gender			<0.001
Male	578 (37.0%)	5,459 (44.9%)	
Female	983 (63.0%)	6,699 (55.1%)	
Year of Diagnosis			0.12
2003	174 (11.2%)	1,207 (9.9%)	
2004	160 (10.3%)	1,239 (10.2%)	
2005	170 (10.9%)	1,161 (9.6%)	
2006	162 (10.4%)	1,134 (9.3%)	
2007	144 (9.2%)	1,122 (9.2%)	
2008	131 (8.4%)	1,162 (9.6%)	
2009	130 (8.3%)	1,137 (9.4%)	
2010	137 (8.8%)	1,088 (9.0%)	
2011	148 (9.5%)	1,066 (8.8%)	
2012	123 (7.9%)	1,035 (8.5%)	
2013	82 (5.3%)	807 (6.6%)	
Age at Diagnosis			<0.001
<65	243 (15.6%)	784 (6.5%)	
65-69	354 (22.7%)	2,160 (17.8%)	
70-79	555 (35.6%)	4,750 (39.1%)	
80-89	345 (22.1%)	3,767 (31.0%)	
>90	64 (4.1%)	697 (5.7%)	
Location of Pancreatic Tumor			0.07
Head of Pancreas	1,111 (71.2%)	8,803 (72.4%)	
Body/Tail	118 (7.6%)	738 (6.1%)	
Unknown	332 (21.3%)	2,617 (21.5%)	
SEER Demographic			<0.001
Northeast	280 (17.9%)	2,695 (22.2%)	
Southeast	691 (44.3%)	2,634 (21.7%)	
Midwest	241 (15.4%)	1,355 (11.1%)	
West	349 (22.4%)	5,474 (45.0%)	
Stage of Disease (AJCC), 6th edition*			<0.001
Stage I	127 (9.2%)	965 (8.8%)	
Stage II	339 (24.4%)	3,030 (27.7%)	
Stage III	140 (10.1%)	1,112 (10.2%)	
Stage IV	538 (38.8%)	3,639 (33.2%)	
Unknown	243 (17.5%)	2,205 (20.1%)	
Charlson Comorbidity Index			<0.001
0	387 (30.5%)	3,716 (39.4%)	
1	330 (26.0%)	2,466 (26.1%)	

≥ 2	552 (43.5%)	3,258 (34.5%)	
Jaundice			<0.001
Yes	203 (13.0%)	2,734 (22.5%)	
No	1,358 (87.0%)	9,424 (77.5%)	
Cholangitis			0.007
Yes	19 (1.2%)	277 (2.3%)	
No	1,542 (98.8%)	11,881 (97.7%)	
ICU Stay After Diagnosis			0.001
Yes	122 (7.8%)	701 (5.8%)	
No	1,439 (92.2%)	11,457 (94.2%)	
Gastric Outlet Obstruction			0.04
Yes	12 (0.8%)	169 (1.4%)	
No	1,549 (99.2%)	11,989 (98.6%)	
